# Knowledge of Errors in the Teaching-Learning Process of Judo-Techniques: Osoto-Guruma as a Case Study

**DOI:** 10.2478/hukin-2014-0053

**Published:** 2014-07-08

**Authors:** Iván Prieto, Alfonso Gutiérrez-Santiago, Miguel Ángel Prieto Lage

**Affiliations:** 1 Faculty of Education and Sports Sciences, University of Vigo, Spain.; 2 Instituto de Investigaciones Marinas (IIM-CSIC), Spain.

**Keywords:** Feedback, martial arts, observation instrument, knowledge of performance, teaching, T-patterns

## Abstract

The aim of this article was to suggest some changes in the teaching-learning process methodology of the judo osoto-guruma technique, establishing the action sequences and the most frequent technical errors committed when performing them. The study was carried out with the participation of 45 students with no experience regarding the fundamentals of judo (21 men and 24 women; age=24.02±3.98 years old) from the Bachelor of Science of Physical Activity and Sport Science at the University of Vigo. The proceeding consisted of a systematic observation of a video recording registered during the technique execution. Data obtained were analyzed by means of descriptive statistics and sequential analysis of T-Patterns (obtained with THEME v.5. Software), identifying: a) the presence of typical inaccuracies during the technique performance; b) a number of chained errors affecting body balance, the position of the supporting foot, the blocking action and the final action of the arms. Findings allowed to suggest some motor tasks to correct the identified inaccuracies, the proper sequential actions to make the execution more effective and some recommendations for the use of feedback. Moreover, these findings could be useful for other professionals in order to correct the key technical errors and prevent diverse injuries.

## Introduction

Judo is one of the most important martial art sports practiced in the world (Parkkari et al., 2004). It includes a significant number of techniques involving changes of direction, twisting, lifting, and landing from falls and throws (Kujala and Taimela, 1995). The analysis of the fundamental performance aspects of a movement can help the instructors to prevent some circumstances that may cause different types of injuries (minor, major and chronic). It can also guide them for increasing the technical effectiveness during the judo combat and they can use it to design precise task-oriented feedbacks.

In this respect, the requirements for the improvement of the technical and tactical preparation have been the focus of some studies which achieved meaningful results with theoretical and practical applications for judo coaching (Adam et al., 2012; Sterkowicz et al., 2013). As an example of the influence that the biotype of judokas has on the technical efficiency, several authors (Melo et al., 2012) have investigated some throwing techniques such as harai goshi and o-soto-gari. They found that in Harai goshi (a hip technique frequently used in judo competitions), the mechanical efficiency in angular displacement was higher and the throw time shorter when uke was taller than tori. With regard to o-soto-gari, better efficiency of performance seemed to be obtained when uke was shorter than tori. Currently, there is a need for developing and applying tools with the general objective of evaluating mechanical efficiency of individual or in-group techniques.

Lack of comprehension of the relation between a poor technique performance and the circumstances that may cause different forms of injuries (minor, major and chronic) has also recently raised a debate (Salanne et al., 2010; Kamitani and Nimura, 2013). In this sense, the experts emphasize that most of the severe injuries, such as head and neck injuries, take place when performing the throw techniques such as o-sotogari (Rodriguez et al., 1998; James and Pieter, 2003; Kamitani and Nimura, 2013. The use of proper techniques and mechanisms is known to be very important in judo. However, there are few publications (Barsottini et al., 2006; Abbott et al., 2008) on the relationship between the different technical aspects and the number of injuries caused. Moreover, more efficient tools are necessary to uncover hidden erroneous sequences of judo techniques, in order to help professionals correct the key technical errors and thus prevent injuries.

Many injuries are attributed to repetitive actions performed with a poor technique. These actions can bring about an excessive pressure on particular joints or muscles contributing to an injury.

Finally, the feedback given by the trainees (Hodges and Franks, 2002) and the use of an appropriate modeling (Zubiaur, 2005) are factors that significantly contribute to the teaching-learning process in both sport and physical education (Zabala et al., 2009; Pereira et al., 2010). This entails detailed knowledge of the key factors to run a motor task, as well as of the most common errors and their sequence. The information provided by professionals is usually based on their personal experience, which results in a pseudoscientific analysis. This lack of knowledge regarding typical errors and their sequences (key points of the projection techniques) may occasionally cause a deficient teaching-learning process. The use of observational analysis and scientific methods to manage the information contributes to eliminate this bias, optimizing the feedback mechanisms. The utility of different feedback methods in the learning process has been compared in previous studies (Reo and Mercer, 2004; Tzetsis et al., 2008). All of them used a detailed examination of the movements as a common approach.

Therefore, the present research is intended to assist coaches and teachers of judo, showing the behavior patterns (errors and their sequences) hidden from visual perception. With the aim of achieving the technical understanding necessary to improve the teaching-learning process, we performed a systematic observational study. In this particular case, the most relevant errors and sequences of errors of the osotoguruma judo technique have been studied. The findings allow to propose some methodological recommendations in the development of tasks. The feedback of the process can be useful for performing the osoto-guruma throw efficiently and for helping professionals to correct the key technical errors, avoiding diverse injuries.

## Material and Methods

In this research, we used an observational methodology (Anguera and Jonsson, 2003) which provides the rigor and flexibility required to study the behavior episodes occurring naturally during the teaching-learning process in judo. Based on Borrie et al. (2002) the type of observation that we undertook was systematic, open and non-participating.

### Design

The observational design (Anguera et al., 2001) was nomothetic (several participants performing the same technique, in this case osotoguruma), based on monitoring (we monitored a throw technique for five academic years), and multidimensional (different dimensions of the observation instrument). According to this design, nomothetic/monitoring/multidimensional (N/M/M), we took some decisions concerning the participants, observational and recording instruments, and analysis procedure.

### Participants

Novice students (n=45; 21 males and 24 females; age 24.02±3.98 years old) were filmed while following a subject of judo at the Faculty of Sciences of Education and Sport at the University of Vigo. The recordings were made during the course of five academic years (2003 to 2008), with the written authorization of the participants that the videos could be used for research purposes. All the ethical standards throughout the study were in accordance with the American Psychological Association (APA).

### Observational instrument

The observational instrument developed for this study was the SOBJUDO-OSGU (Table 1), combining the field format with the category system (Fernández et al., 2009). Technical errors of judo techniques (which are the aim of our study) are included as one of the criteria of this observational instrument. The methodological model used for the teaching-learning process of both performance and observation was based on the indications provided by Kodokan (Kodokan, n.d.) school.

The SOBJUDO-OSGU instrument fits the observational design presented. Thus, it is multidimensional in nature including the following structural criteria: grip, off-balance, right-foot position, right-arm position, hip position, left-foot position, leg′s action, blocking action, throw stage, control stage and globality. Each dimension gives rise to a system of categories that meets the conditions of exhaustiveness and mutual exclusivity (EME).

### Recording instrument

The data collection was performed by recording the student from two different angles with two digital cameras (JVC GZ-MG21E). To assist the analysis of the projections recorded, the filmed material was edited with the software Pinnacle Studio v.12. The software Match Vision Studio Premium v.3.0. (Castellano et al., 2008), a multimedia interactive program that allows simultaneously viewing and registering the filmed material in a computer, was used to systematically support the observational analysis. This program is highly flexible, allowing us to introduce all the codes for each of dimensions of the SOBJUDO-OSGU variable criteria, in order to register its occurrence.

### Procedure

The technical execution of osoto-guruma was filmed all along the ordinary training period (∼4 months with 3 hrs of practice per week) at the University of Vigo, involving the learning process of a total of 17 projections. We only used the data collected from 10 of the techniques. The selection criteria of the techniques studied were based on: 1) the premises of an investigation reporting the performance difficulty of certain techniques of Gokyo (García et al., 2009); and 2) a questionnaire addressed to the faculty members who, at that time, were teaching at the Faculty of Sport Science the subject of “fundamentals of learning judo-techniques”. In both cases, the osoto-guruma projection was considered one of the 10 easiest techniques to learn. The filmed material was taken by recording each participant when performing the techniques they had learnt, without opposition and from a static position (technical), employing a stratified random sampling. After observation and recording of all the technical actions, we obtained an Excel file for each projection with its sequential record.

The quality control of the data recorded by two observers was performed by means of the Cohen’s Kappa (Cohen, 1968) coefficient (k), that guarantees the agreement between them when the value k is >0.80. The software GSEQ (Bakeman and Quera, 2001) provided this statistical test. The value obtained for our study was k=0.85. Once the quality control test was completed, it was possible to carry out a first descriptive analysis of the frequencies and percentages of appearance of technical errors.

The excel files obtained allowed to have the frequencies of all the codes of occurrences registered, which were successively transformed in order to enable further analyses. The codes of the instrument of observation SOBJUDO-OSGU were entered into the software THEME (Magnusson, 2000, 2005), a computer application extremely effective for the study of team and individual sports (Fernández et al., 2009; Gutiérrez-Santiago et al., 2011), with the aim of detecting the temporal patterns. The temporal patterns (T-Patterns) obtained using the algorithm present in the THEME v.5 (Magnusson, 2000) served to reveal the hidden structures and unobservable aspects of osoto-guruma technique.

### Data analysis

The frequency of occurrence of the different errors committed during the osotoguruma throw performance was determined by means of a descriptive analysis using SPSS 15. The results of this analysis are shown in Table 2. An analysis of temporal patterns among the observed errors was also conducted using THEME. The aim of this analysis was to identify the most significant error sequences. To perform the qualitative analysis we estimated the coincidence among the observers using the Cohen’s Kappa (Cohen, 1968), obtained with the GSEQ (Generalized Sequential Querier) software.

## Results

### Statistical analysis

In Table 2 a descriptive analysis of the errors found in the study group (n=45) is presented.

The most common errors are those related to the initial imbalance (NOB), the improper position of the supporting foot (IPSF) and the incorrect blocking action (SF), with a deficient traction action and an incorrect direction of the arms accompanied by an insufficient rotation of the trunk at the end of the technique (IAT).

### Detection of temporal patterns

**Figure 1:**
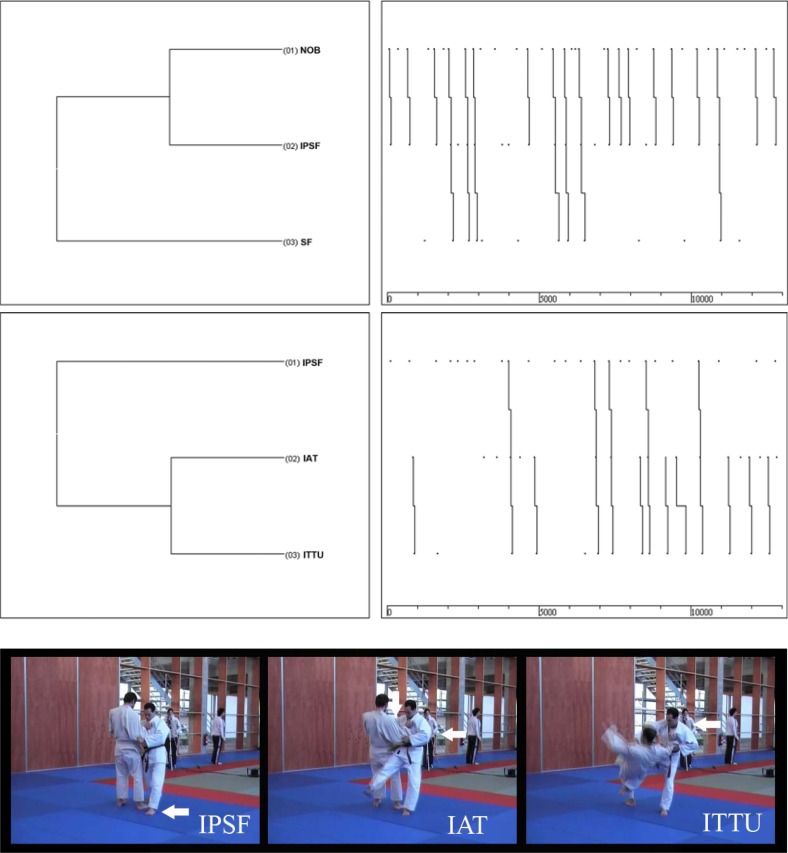
First and second osoto-guruma dendrogram

In Figure 1, we show the sequence of errors detected. The left quadrant represents the relationship established between the different categories (in this case the different technical errors, see observational instrument of SOBJUDOOSGU). Its reading must be carried out as a tree diagram from left to right. The right quadrant allows us to determine how often the previous relationships take place using the lines that go from the top to the bottom.

Figure 1 shows a strong link between the incorrect initial imbalance (NOB), the inappropriate positioning of the foot (IPSF) and the entire support of the right foot on the ground with the consequent incorrect distribution of the body mass between both legs (SF) during the intermediate phase of the technique. Similarly, this incorrect placement of the supporting foot (IPSF) is usually bound to an inadequate traction action of the arms (IAT) and an insufficient rotation of the trunk (ITTU) at the end of the projection.

**Figure 2:**
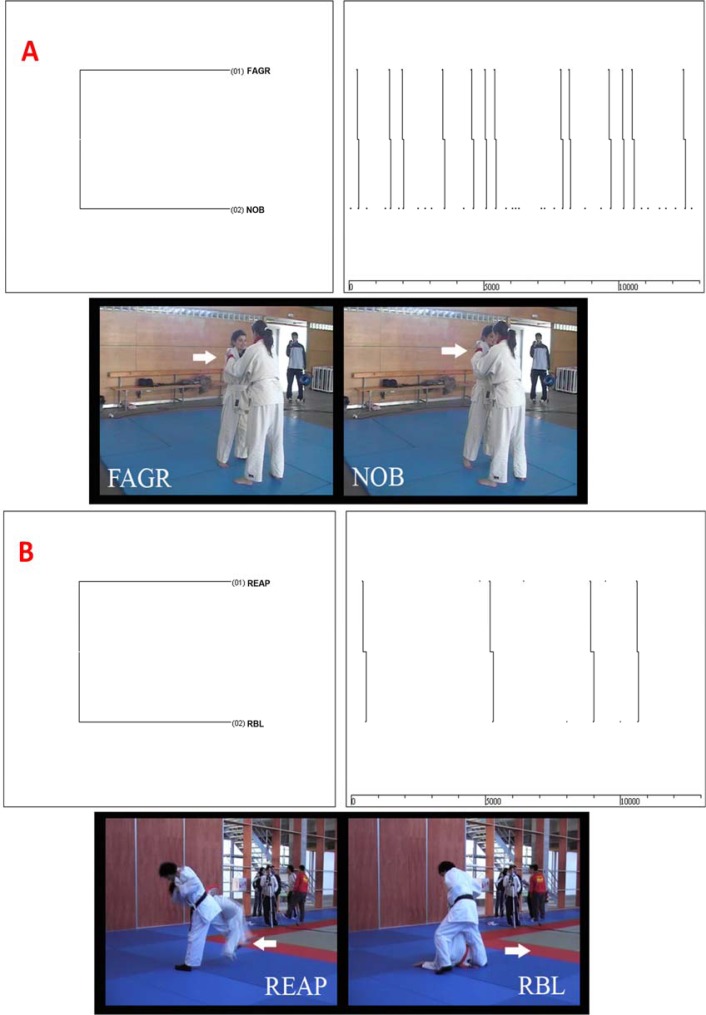
Sixth and Ninth osoto-guruma dendrograms

Figures 2a and 2b show other important dual relationships obtained in the sequential analysis of the *osoto-guruma* technique. Thus, in Figure 2 we can observe that an erroneous first grip is usually preceded by an ineffective imbalance of the adversary (FAGR-NOB). Similarly, improper sweep action forces *tori* to replace the leg previously used to perform the sweeping (REAP-RBL) (in *osoto-guruma,* a blocking action must be performed).

## Discussion

After a thorough revision of the literature, we have confirmed the lack of scientific studies regarding the technical errors and its sequences in judo. Only the most relevant authors of this field of study have pointed out the key aspects or the most common technical errors when describing a technique (Mifune, 2004; Daigo, 2005; Ohlenkamp, 2006). Such indications, mainly based on their personal and professional experience, persistently agree with our findings. This fact demonstrates the reliability of the method applied in our study.

Thus, several authors have highlighted that the imbalance of the opponent at the beginning of the technical action must be directed towards the back right diagonal (NOB) and tori must make uke be balanced only by the heel of his right foot (Mifune, 2004; Daigo, 2005; Ohlenkamp, 2006; Taira, 2009).

The incorrect placement of tori’s supporting foot in the second and third phases of the projection (IPSF) is one of the typical errors observed, what has also been mentioned by other authors (Ohlenkamp, 2006; Taira, 2009). According to the technical model, the placement of tori’s supporting foot must be parallel to the opponent’s right foot (Mifune, 2004) or slightly forwards but no more than 10 cm (Daigo, 2005).

Another common error detected is the incorrect distribution of tori’s mass on legs at the time of throwing the opponent. The most correct approach would be to lean the body mass on the leg that does not participate in the blocking action (SF and WLB). However, there is a tendency to poise the right foot fully on the floor. Although nobody has specifically mentioned this aspect as a typical error or a fundamental point, some authors state that the body mass should entirely fall on the left leg (Taira, 2009).

Additionally, some individuals perform a sweeping action instead of performing the blocking action (REAP). Some authors, such as Inogai and Habersetzer (2002), specifically stated that the difference between this technique and osoto-gari is the action conducted with the right leg. In osoto-guruma there should be no sweeping action, but the right leg must be used to block the opponent’s body.

Several authors, such as Ohlenkamp (2006) and Uzawa (1981), have also highlighted in their observational analysis that the insufficient action of the arms (IAT) is a frequent error.

The sequential pattern analysis of errors reveals that an improper imbalance of uke leads to inaccurate positioning of the body, preventing tori from distributing body mass in a suitable way to perform the projection. That makes it difficult to carry out an optimal action with the arms and to perform the body rotation towards the right direction. Few references were found regarding the pattern of this chain of errors and, to our knowledge, the connection between the major errors observed in osoto-guruma judo-technique had not previously been explained in detail.

Several authors have reported some dual relationships between errors. Taira (2009) indicated that, in order to make the initial imbalance, it is necessary to perform the grip properly (FAGR-NOB), a relationship observed in one of T-Patterns obtained. Mifune (2004) emphasized the importance of placing the mass of the opponent on their heels during the imbalance, thus the performer can easily put the supporting foot outside of the location of the uke’s right foot (NOB-IPSF). In this sense, the link between the RBL and REAP errors has not been described, mainly because osoto-guruma only involves a blocking action. However, when looking at the considerations of other authors about osoto-gari (technique in which a sweeping action must be performed), authors (Yamashita, 1993) indicate that an action can affect the later performer’s rebalancing due to its own inertia.

### Study limitations and future perspectives

One of the limitations of this study was the time available for students to practice (four months), as well as the nature of the participants (university students). Therefore, future research employing longer learning periods (>10 months), other origins of the participants (high school, judo clubs, etc.) or different age groups could be interesting. The process of learning judo techniques could possibly be better analyzed this way, establishing guidelines and setting the practice time required to identify the most common mistakes. Further studies could also be undertaken in order to identify the factors influencing the number and importance of errors and their sequential registration.

It is a matter of fact that the teaching and learning process of osoto-guruma technique could be improved by paying special attention to the following statements regarding the movement sequences, which will ensure that the throw is correctly executed:

1. A proper unbalance of the opponent’s body promotes the correct location of the supporting foot.
2. A proper placement of the supporting foot and a suitable distribution of the body mass simplify the subsequent blocking action on the uke’s body and the correct execution of the projection phase of osoto-guruma technique.

### Practical implications based on knowledge of performance (KP)

The results of this study also enable us to suggest a number of strategies, based on the knowledge of performance, with the aim of improving the teaching and learning of the osotoguruma technique.

When demonstrating the technique, the student should pay attention to the key points highlighted in this study. As far as the throw theoretical aspects are concerned, we think that coaches could find it useful to incorporate some video-recordings or other images illustrating the fundamental features and the common errors detected here. In any case, teachers or coaches should only focus on the most relevant aspects.

Instructors could design tasks or drills that draw the student’s attention to the most significant errors and sequences of behavior detected.

After a throw execution in the training, the subsequent communication between coaches and students could be improved by providing a more precise feedback. Coaches should firstly consider the most significant errors and sequences identified in the present study, leaving others for a later stage of the training. It would also be helpful to take into consideration just a few key aspects to avoid overloading the students with an excess of information. The results of this study can provide a platform for different kinds of feedback (verbal, verbal with a practical demonstration or verbal with hands-on assistance), which should always be positive in nature.

Coaches could elaborate some observation/evaluation sheets based on the category system of the observation instrument used in this study. One model should be intended for the students to work in groups of three, with one of the trainees observing the other two while they are performing the throw. Thus, this student would conduct an observational analysis using the evaluation sheet, noting the errors made and providing an immediate feedback. The same observational analysis could also be carried out later by means of video recordings.

## Figures and Tables

**Table 1:** Observational instrument of SOBJUDO-OSGU

**CRITERION**	**CODE**	**DESCRIPTION**
GRIP	FAGR	While performing the technique, *tori* (the person executing the action) grips the *judogi* of *uke* (the person who is thrown) with his/her left hand, around the midpoint of the forearm.
OFF-BALANCE	NOB	*Tori* does not put *uke* off-balance in the first part of the technique. His arms maintain the initial grip and only serve to accompany the action.
	DOB	The frontal off-balancing action and the subsequent initial displacement are performed in a discontinuous way.
POSITION OF SUPPORTING FOOT	IPSF	After the initial movement *tori* places his/her left foot incorrectly. The supporting foot is either too far forward or too far back. The correct position is parallel to and aligned with *uke’s* right foot.

INCORRECT BLOCK PLACEMENT	IBP	When *tori* places his right leg behind *uke’s* left leg, *tori* makes an inappropriate placement of his leg in one of the following ways: 1) it lies behind *uke’s* left leg (about 15 cm) without contact between the two legs; 2) he only contacts with his right ankle on *uke’s* left ankle; or 3) the contact that occurs between *tori* and *uke* is only in the twin.
	KN	The right knee joint of *tori* is flexed during blocking action.
BLOCKING ACTION	NBLC	*Tori, during the execution of the technical action, does* not block at any time the *uke’s* body on his leg.
	REAP	*Tori*, instead of blocking the body of *uke*, performs a sweeping action with his right leg.

FINAL BLOCKING PHASE	WLB	Part of the body weight of *tori* during the blocking action is leant on his right leg.
	BANS	The execution of the block of the leg and the arm action over *uke* is performed with continuity.
	SF	*Tori* entirely supports the sole on the ground with the foot used to carry out the blocking action, keeping also part of the body weight on it.

THROW STAGE	IAT	During the final stage of the throw, *tori’s* arms produce an insufficient force when throwing *uke’s* body to the floor.
	ITTU	*Tori* fails to turn his trunk enough in the guiding stage of the technique.

CONTROL STAGE	FACC	During the guiding stage *tori* uses his/her right arm to accompany *uke’s* fall to the floor.
	FNC	During the final stage of the technique *tori* performs no action with his/her left hand and therefore fails to control the fall of the adversary’s body.
	KTB	During the final stage of the throw *tori* bends his/her trunk around 90º with respect to the vertical plane, maintaining this position once the throw is complete.

REBALANCING	RRF	After performing the throw *tori* loses his/her balance. In order to regain it he/she steadies himself with his/her right foot.
	RLF	Upon completion of the technique *tori* loses his/her balance, which he/she regains by steadying him-/herself with his/her left foot.
	RRH	After throwing the adversary *tori* loses his/her balance in a forward direction (sagittal plane) and uses his/her right hand to correct his/her position.
	RBL	After the blocking action *tori* uses the leg that performed the reap to regain balance.
GLOBALITY	SLEX	The throw is executed slowly and without any continuity.

**Table 2:** Frequency and percentage of occurrence of technical errors made when performing the osoto-guruma

	**ERROR**	**FREQUENCY**	**PERCENTAGE**
GRIP	FAGR	13	28,9%

OFF-BALANCE	NOB	36	80%
	DOB	3	6,7%

POSITION OF SUPPORTING FOOT	IPSF	25	55,6%

INCORRECT BLOCK PLACEMENT	IBP	3	6,7%
	KN	1	2,2%

BLOCKING ACTION	NBLC	1	2,2%
	REAP	7	15,6

FINAL BLOCKING ACTION	WLB	3	6,7%
	BANS	2	4,4%
	SF	13	28,9%

THROW STAGE	IAT	19	42,2%
	ITTU	15	33,3%

CONTROL STAGE	FACC	9	20%
	FNC	5	11,1%
	KTB	2	4,4%

REBALANCING	RRF	1	2,2%
	RLF	4	8,9%
	RRH	2	4,4%
	RBL	6	13,3%

GLOBALITY	SLEX	2	4,4%
